# An Unusual Presentation of Lupus in a Pediatric Patient

**DOI:** 10.1155/2013/180208

**Published:** 2013-08-27

**Authors:** Vimal Master Sankar Raj

**Affiliations:** Children's Hospital of Illinois, OSF Medical Center, Division of Pediatric Nephrology, Department of Pediatrics, 530 NE Glen Oak Avenue, Peoria, IL 61637, USA

## Abstract

Systemic lupus erythematosus (SLE) is an autoimmune disease causing inflammatory tissue damage. Multiple organ damage can ensue with renal and neurological involvement carrying the worse prognosis. In this case report we present a 10-year-old African American girl who presented with abnormal choreiform movements, headache, weight loss, and fatigue. Detailed clinical examination with laboratory and imaging studies clinched the diagnosis of SLE. Echocardiogram revealed the presence of Libman-sacks endocarditis. Patient showed rapid resolution of symptoms with steroid therapy. A brief discussion on childhood onset lupus along with the varied clinical presentation is discussed.

## 1. Case Report

A 10-year-old African American girl was admitted to our hospital with chief complaints of fever and abnormal movements.

The patient is a previously healthy, a 10-year-old girl who two weeks prior to admission started having episodes of headache on and off controlled with Tylenol. Headache was generalized, not associated with nausea or vomiting. Three days prior to admission, the patient had low grade fevers, and mom noticed some abnormal movements of the extremities which progressed over the next 2 days to the point where she cannot eat or dress on her own. She also had difficulty in walking and holding on to objects. H/o lip smacking and slurred speech are present for the past 3 days. H/o rash in the lower extremities which looked like hives is present for the past 2 days.

H/o decreased appetite with loss of weight is present. The day prior to admission the patient had fevers with temperature up to 101 F, and with progression of involuntary movements the patient was taken to her PCP who started her on Acyclovir. With the symptoms getting worser, the patient was taken to an emergency center that evening before being transferred to our hospital for further care and management. 

No H/o any prior hospitalization. Past H/o sore throat about a month ago which lasted for 2 days. At that time the child did not receive any medical attention, and sore throat went away on its own.

The child was adopted, and not much is known about the birth and family history. As per adoptive parents biological mom might have been worked up for some autoimmune diseases, the specificity of which is unknown.

On admission, the patient had a temperature of 99.5 F, weight of 55.5 kg (lost 10 pounds over the past 2 to 3 weeks), blood pressure of 99/77, heart rate of 129, and respiratory rate of 20.

On Neurological exam, the patient was awake, alert and cooperative. Involuntary lip smacking with tongue protrusion and slurred speech were present. Pronounced choreoathetoid movements of the upper and lower extremities were present. Milkmaid grip was positive. With arms outstretched above the head noticeable chorea with pronation of forearm was present. 

On Motor exam Tone and reflexes were equal and normal in all 4 limbs. Strength was decreased in the lower extremities and choreiform movements were present. 

Sensation was intact. She had trouble with walking without support.

She had a diffuse macular rash involving the lower extremities which is erythematous and confluent in some areas. The rest of her physical exam including her thyroid, cardiovascular, respiratory, and abdominal exam was normal.

Lab values from the outlying ER include a CBC which showed anemia with a Hb of 10.8 g/dL, hematocrit of 32.5%, MCV of 76.5, and thrombocytopenia with platelet count of 89000/mm^3^. CT scan and a spinal tap were done which were essentially normal. CSF studies showed no pleocytosis with normal protein and glucose. A gram stain of the CSF was negative. Bacterial culture and HSV PCR were pending at the time of admission.

### 1.1. Course in the Hospital

The patient was worked up for the differential of choreiform movements. High on the list of our suspicion was sydenham's chorea secondary to rheumatic fever. ASO titre was elevated at 215 IU/ML (<150), ESR was elevated at 55 mm/hr (0–10), and urine analysis was positive for proteins at 25 mg/dL (neg), ketones at 50 mg/dL, and small blood. The patient was initially started on penicillin.

**Figure 1:**
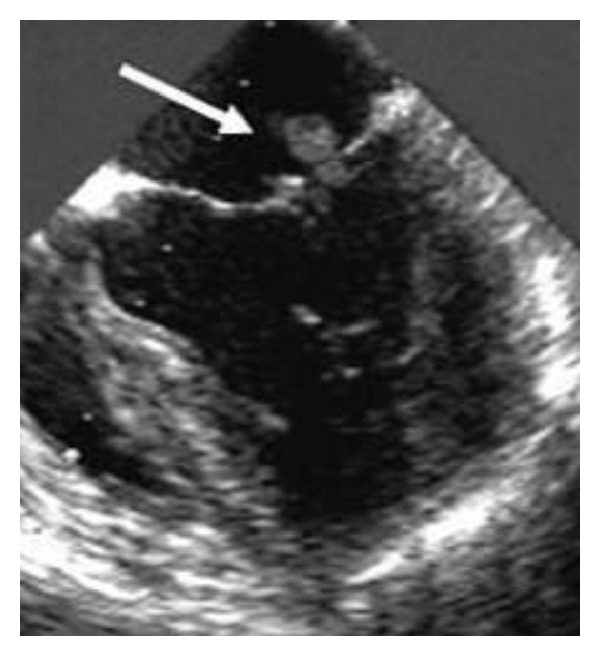
Trans esophageal echocardiogram showing Libman-sacks vegetation.

An echocardiogram of the heart was done (Figure 1) which showed 9 mm × 8 mm echogenic, nonmobile area on the posterior mitral valve leaflet suspicious for thrombus versus vegetation with normal function, and anatomy of the valves.

MRI of the brain was done which was normal except for nonspecific small white matter hyperintensities in the left frontal lobe.

With the patient's history of fever, and possible thrombus versus vegetation on echocardiogram, thought of infective endocarditis was entertained. Blood cultures were sent, and the patient started on Lovenox and ceftriaxone pending culture results. For the possible thrombus, coagulation workups were done including a prothrombin time which was normal. Test for Cardiolipin IgG was negative at 8.3 GPL (Neg < 10.0 GPL) and Borderline positive for Cardiolipin IgM at 14.5 GPL (Borderline 10.0–14.9 GPL).

Next on the list of our differential was systemic lupus erythematosus. With Biologic mom's possible workup for autoimmune disease and the fact that chorea even though is a rare complication for SLE can be a presenting feature especially in pediatric SLE, a blood analysis for ANA was sent. Other labs that were done to rule out other causes of chorea including urine and serum toxicology screen, thyroid panel, and lyme titres which came back negative. 

On day 2 of the hospital course ANA titres came back positive at 1 : 1280 with a homogenous pattern. Further testing for SLE revealed positive results for anti-DS DNA at 473 IU/ML (Neg < 100), anti-Smith antibodies at 177AU/mL (Neg < 100), Sjogren's anti-SS-A at 937 AU/mL (Neg < 100), Sjogren's anti-SS-B at 118 (Neg < 100), and antihistone abs at 330 AU/mL (Neg < 100), and complement levels for C3 was low at 55 mg/dL (86–184). CPK levels were also abnormally elevated.

Blood cultures and HSV PCR from the outlying hospital came back negative. Ceftriaxone and acyclovir were stopped and patient was started on IV steroids for SLE.

The patient's chorea dramatically improved after starting steroids. She received Solu-Medrol at 500 mg daily for 3 days and slowly tapered over to oral steroids over a course of 2 weeks. The patient was discharged home on oral steroids and Lovenox with follow-up appointments scheduled with rheumatology and cardiology.

## 2. Discussion

### 2.1. Systemic Lupus Erythematosus

Systemic lupus erythematosus is an autoimmune disease characterized by the formation of antibodies against self-antigens leading to inflammatory tissue damage. Though lupus predominantly affects women in the reproductive age group, incidence in childhood varies between 10% and 20% depending on various studies [1–3].

### 2.2. Childhood Onset SLE

Mean age of onset for childhood SLE is between 11 and 12 years [1, 2] and more prevalent in black, Hispanic, and Caucasian girls in descending order [3]. Most common presenting features for pediatric SLE include fever, nephropathy, and lymphadenopathy [2] with more active disease during presentation as well as followup [4]. Children because of their more active disease status end up getting much more intensive drug therapy with steroids and cytotoxic agents when compared to adults.

### 2.3. Cardiac Manifestations of SLE

The effect of SLE on the cardiovascular system is diverse and includes pericarditis, myocarditis, endocarditis, and coronary artery disease secondary to atherosclerosis or arteritis. Among these valvular disease is the most prevalent and a very common cause for morbidity in SLE patients. Manifestations include leaflet thickening, valvular stenosis, or regurgitation with vegetations or thrombi on the valve surface.

Mitral valve is the most commonly involved valve with leaflet thickening as the most common abnormality followed by vegetations [5]. 

### 2.4. Libman-Sacks Endocarditis

Libman-sacks endocarditis was first reported in 1924 as a bacterial free verrucous vegetation of the valves. Its prevalence in SLE varies and has been found as high as 60% in autopsies. Studies using Doppler echo for the prevalence of Libman-sacks vegetation in a large cohort of SLE patients have shown an incidence of 1 in 10 [6]. The vegetations are found predominantly in the mitral valve mostly on the atrial surface [5]. 

### 2.5. Pathogenesis

The vegetations in Libman-sacks are thought to be due to deposition of fibrin platelet thrombi on the valve surface which organizes and causes fibrosis. Immunologic injury to the valves as demonstrated by Shapiro et al. with deposition of immunoglobulins and complement [7] might mark the initial insult that attracts the platelet fibrin thrombi.

### 2.6. Significance of Libman-Sacks Endocarditis

The significance of Libman-sacks endocarditis in disease progression of SLE is not well understood. Even though initially thought off as an incidental autopsy finding, studies have shown association between presence of Libman-sacks vegetation and progression of valvular dysfunction and a tendency towards thrombotic events [6]. The association of Libman-sacks vegetation with antiphospholipid antibodies has been documented in several studies involving large cohort of SLE patients [6, 8, 9] and might account for the higher incidence of thrombotic events.

### 2.7. Neuropsychiatric Manifestations of SLE

SLE-related central nervous system features have a wide spectrum of presentation ranging from mild cognitive defects to overt neuropsychiatric features including stroke, seizures, chorea, anxiety, depression, and acute psychosis.

Neuropsychiatric features though not so common in adult population are much more prevalent in Juvenile onset SLE [10, 11] with headache and seizures being the most common presenting features.

### 2.8. Chorea in SLE

Chorea is a well recognized but rare complication of SLE. Case reports of chorea as a presenting feature of SLE have been documented in the literature [12, 13]. The prevalence of chorea is variable with different studies and can account for 2% to 6% of neurological manifestations in SLE [10, 11]. 

### 2.9. Pathophysiology

Pathophysiology of chorea is not well understood. Currently 2 theories have been hypothesized.

First theory is based on the observation of higher incidence of antiphospholipid antibodies in patients with chorea. Reversible injury to the basal ganglia by these antibodies might lead to chorea [14].

The next hypothesis is based on the presence of antineuronal antibodies in the CSF of patients with neurologic manifestations. An association between antiribosomal P protein antibodies in CSF and neuropsychiatric syndromes in SLE has been documented in some studies [15–17].

### 2.10. Role of Antiphospholipid Antibodies

Antiphospholipid antibodies are a group of autoantibodies against phospholipid and phospholipid binding proteins. The antibodies include anticardiolipin antibodies, anti-*β*_2_ glycoprotein antibodies, and lupus anticoagulant. The association of these antibodies with thrombosis constitute antiphospholipid syndrome.

APL antibodies are found more commonly in Juvenile onset SLE [2] and also in association with Libman-sacks endocarditis and chorea and represent a risk for vascular events like thrombosis, myocardial infarction, and stroke [9].

### 2.11. Role of Anticoagulation

In patients with antiphospholipid antibodies, a thrombotic event secondary prevention with high dose warfarin maintaining an INR > 3 has been recommended [18]. But for primary prevention of thrombotic events whether low dose aspirin can help is a question yet to be answered with conflicting reports in various studies [19]. Complete resolution of Libman-sacks vegetation after initiation of warfarin therapy has been documented [20, 21].

### 2.12. Prognosis in Childhood Onset SLE

Childhood onset SLE has a grimmer prognosis when compared to adult onset SLE. Even though 10 year survival rates for pediatric SLE has improved over the last decade ranging between 80% to 90% these children end up suffering extensive morbidity because of the longer duration of disease activity causing cumulative damage as well as the side effects from medication [22]. The earlier age of onset also correlates with a more severe disease [2] which can contribute to a worse prognosis as well. The persistent presence of antiphospholipid antibodies is also considered a risk factor for eventual poor prognosis [22].
